# Effect of a 1-year intervention comprising brief counselling sessions and low-dose physical activity recommendations in Japanese adults, and retention of the effect at 2 years: a randomized trial

**DOI:** 10.1186/s13102-021-00360-7

**Published:** 2021-10-25

**Authors:** Julien Tripette, Yuko Gando, Haruka Murakami, Ryoko Kawakami, Kumpei Tanisawa, Harumi Ohno, Kana Konishi, Michiya Tanimoto, Noriko Tanaka, Hiroshi Kawano, Kenta Yamamoto, Akie Morishita, Motoyuki Iemitsu, Kiyoshi Sanada, Nobuyuki Miyatake, Motohiko Miyachi

**Affiliations:** 1 Center for Interdisciplinary AI and Data Science, Ochanomizu University, Bunkyo, Tokyo, Japan; 2 Department of Physical Activity Research, National Institutes of Biomedical Innovation, Health and Nutrition, Shinjuku, Tokyo, Japan; 3 Faculty of Sports Science, Surugadai University, Hanno, Saitama Japan; 4 Faculty of Sport and Health Science, Ritsumeikan University, Kusatsu, Shiga Japan; 5 Faculty of Sport Sciences, Waseda University, 2-579-15 Mikajima, Tokorozawa, Saitama 359-1192 Japan; 6 Faculty of Health Care, Kiryu University, Midori, Gunma Japan; 7 Faculty of Food and Nutritional Sciences, Toyo University, Itakura, Gunma Japan; 8 Faculty of Biology-Oriented Science and Technology, Kindai University, Kinokawa, Wakayama Japan; 9 Research Center of Health, Physical Fitness and Sports, University of Nagoya, Nagoya, Aichi Japan; 10 Faculty of Letters, Kokushikan University, Setagaya, Tokyo, Japan; 11 Faculty of Pharmaceutical Sciences, Teikyo Heisei University, Nakano, Tokyo, Japan; 12 Okayama Southern Institute of Health, Okayama Health Foundation, Okayama, Okayama Japan; 13 Faculty of Medicine, Kagawa University, Miki, Kagawa Japan

**Keywords:** Physical activity, Randomized control trial, Moderate-to-vigorous physical activity, Step-count, Counseling, Health promotion, Cohort study, Accelerometer monitor

## Abstract

**Background:**

In an effort to increase people’s adherence to active lifestyles, contemporary physical activity (PA) guidelines now include low-dose PA.

**Methods:**

PA was evaluated in 583 participants of the Nutritional and Physical Activity Intervention Study (NEXIS) cohort (30–65 years old); 349 inactive participants (MVPA, 2.7 ± 1.0 MET-h/day) were randomly assigned to the intervention or control groups, and 235 active participants participated in follow-up visits. The intervention aimed to increase MVPA and comprised five brief counseling sessions over 1 year. The 1-year target for the participant was increasing their step-count to 10,000 steps/d or +3000 steps/d, relative to the baseline score. The counseling sessions were designed to stimulate progressive changes in physical behaviors by recommendations promoting small and/or light-intensity bouts of PA. PA was measured at baseline, the end of the intervention, and 1 year after the intervention ended. Additionally, several nutrition, health, and fitness parameters were measured.

**Results:**

Participants in the intervention group significantly increased their step-count from 8415 ± 1924 at baseline to 9493 ± 2575 at the end of the 1-year period. During the same period, MVPA significantly increased by 0.9 MET-h. The daily time spent in ≥ 3, ≥ 4 and ≥ 5 MET activities increased by 11, 6, and 3 min, respectively. This increase in PA remained observable 1 year after intervention concluded. The active group maintained higher physical activity levels throughout the two years. The intervention group showed smaller energy intakes at the end of the 2-year period. Significant correlations were noted between the 1-year change in MVPA and the change in resting heart rate (r =  − 0.22), and between the 2-year change in MVPA and the change in waist circumference (r =  − 0.08) and peak oxygen consumption capacity (r = 0.23) in the intervention group only.

**Conclusions:**

A prolonged and progressive PA intervention promoting small bouts of light-to-moderate PA may be used in healthy, not-optimally-active people to increase PA beyond the strict period of the intervention. Further studies are necessary to understand whether low-dose PA messages can be effective in initiating a progressive increase toward larger amounts of PA.

*Trial registration*: Clinical Trials.gov, NCT00926744, retrospectively registered.

**Supplementary Information:**

The online version contains supplementary material available at 10.1186/s13102-021-00360-7.

## Introduction

Numerous studies have described insufficient physical activity (PA) as a risk factor for a wide range of non-communicable diseases, including heart diseases [1], diabetes mellitus [2], cancers [3], pulmonary diseases [4], and dementia [5]. Consequently, physical inactivity is estimated to be annually responsible for > 5 million premature deaths worldwide [6].

In 2010, in an effort to address physical inactivity, the *World Health Organization* (WHO) released PA guidelines [7]. The WHO recommends a minimum of 150 min of moderate-intensity PA or 75 min of vigorous-intensity aerobic PA per week. In Japan, the *Ministry of Health, Labour and Welfare* released the “Physical activity guidelines for the promotion of population health” (or simply “ActiveGuide”) in 2013 [8]. This document recommends that adults under 65 years of age perform 60 min of moderate-to-vigorous PA (MVPA) every day (equivalent to 3.3 metabolic equivalent of task [MET]-h/d). The ActiveGuide includes the “+10” topline message, which stands for “add 10 min of physical activity every day” and points toward several other low-dose PA recommendations. This original top message and content aims to keep the recommended amount of PA to a minimum to increase the chances of adherence, that is, removing barriers for people who may not have sufficient time or intrinsic motivation to engage in prolonged PA on a daily basis [9, 10].

PA can be characterized quantitatively using the three following dimensions: frequency, duration and intensity [11]. One dose of PA corresponds to a volume of PA defined as the product of the frequency, total duration, and mean intensity of the activity bouts occurring over a given period of time. A low dose of PA therefore coincides with a small volume of PA and may result from the low intensity, low duration, or low frequency of the activity bouts, or a combination of these features. The dose–response relationship between PA and mortality is now well documented, even in low-volume regions [9, 12–17]. For instance, a higher daily step-count, even that of < 5000 steps/d, has recently been associated with lower mortality in American adult women [17]. The existence of such a relationship would tend to support the systematic inclusion of low-dose PA messages in official PA recommendations, but the effectiveness of these messages to initiate a progressive increase in PA toward larger volume remains unaddressed in randomized control trials (RCTs).

The present study aimed to comprehensively determine the effectiveness of a PA intervention composed of brief counseling sessions and low-dose PA recommendations in healthy yet inactive Japanese adults. The assessment of a large panel of nutrition, health, and fitness parameters allowed us to further explore the effect of a low-dose PA intervention. More specifically, the following four questions were addressed:

- *Question #1*: Is a 1-year PA intervention composed of brief counseling sessions and low-dose PA recommendations effective in increasing the level of MVPA in a population of healthy Japanese adults?
- *Question #2*: Are active behaviors possibly adopted during this type of PA intervention still observable 1 year after the end of the intervention?
- *Question 3*: Is a 1-year PA intervention composed of brief counseling sessions and low-dose PA recommendations able to yield any significant physical fitness and health benefits?
- *Question #4*: Which factors influence the response to the PA intervention and the retention of the effect beyond the 1-year intervention period?

## Materials and methods

### Protocol overview

The Nutrition and Exercise Intervention Study (NEXIS, Clinical Trials.gov, identifier: NCT00926744) is a multi-year longitudinal cohort study aiming to monitor the association between lifestyle and health markers in the Japanese adult population. In total, 1075 participants were recruited in the Tokyo metropolitan area (N = 819) and Okayama prefectures (N = 256) between 2007 and 2015. A total of 224 participants were excluded from analysis because they were older than 64 years at the time of baseline measurements or because they met at least one of the following exclusion criteria: history of stroke, cardiac disease, or chronic renal failure or difficulty with ambulation due to knee or back pain. The remaining 851 participants underwent an objective evaluation of PA using accelerometer-based activity monitors. Three hundred and fifty-six participants who presented both an MVPA level of ≥ 3.3 MET-h and a step-count value of ≥ 10,000 steps were allocated to the “active” group. The remaining 495 participants were assigned randomly to one of the two following groups: “PA intervention” (N = 248) or “control” (N = 247). After 327 participants, who were referred to the National Institutes of Biomedical Innovation, Health, and Nutrition in Tokyo renewed their consent in 2019, the number of participants allocated to each group was updated as follows: 186 participants in the PA intervention group, 163 participants in the control group, and 234 participants in the active group.

**Fig. 1:**
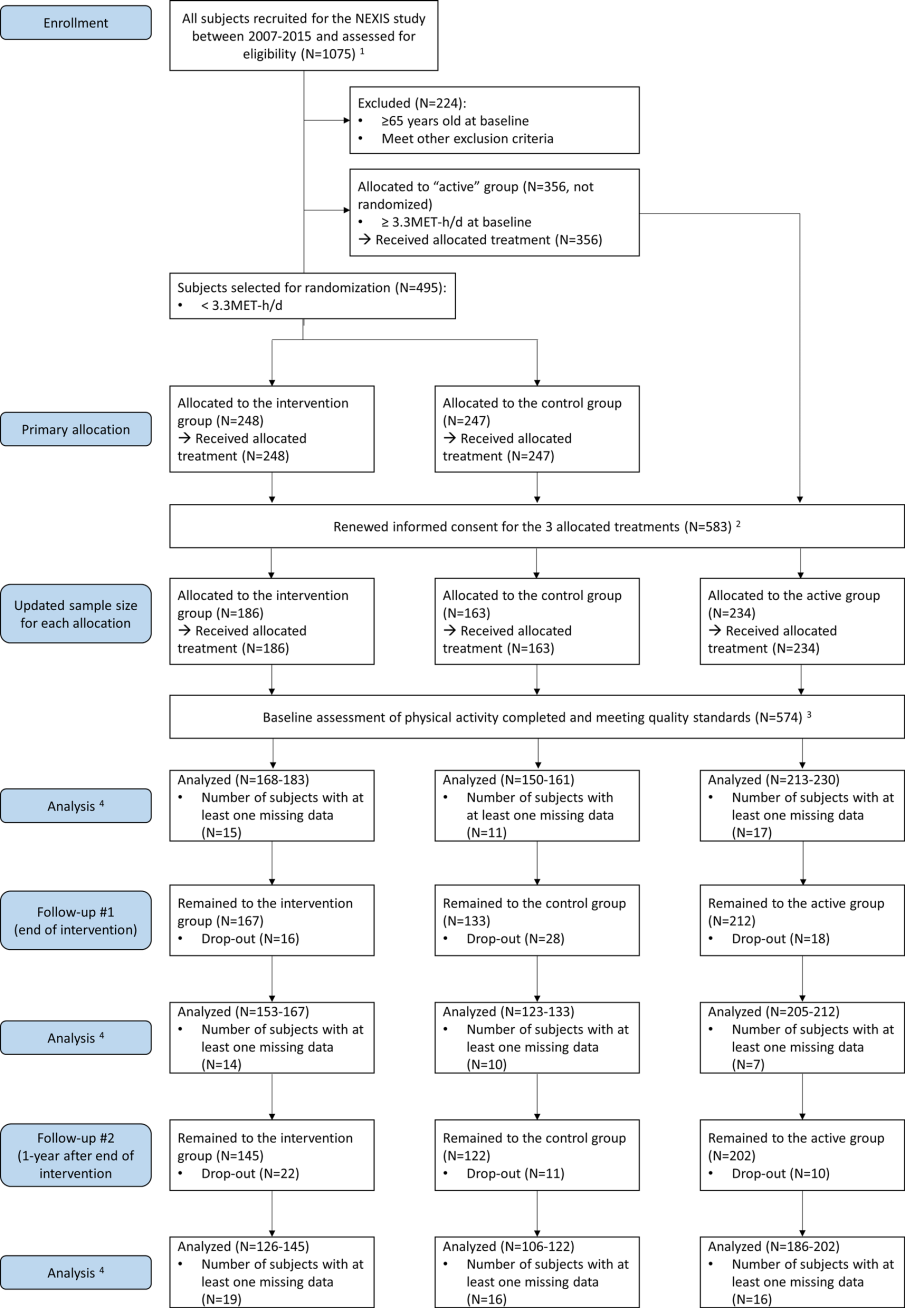
CONSORT flow chart of the study. ^1^Sample size counted in 2015. ^2^Five-hundred-eighty-three of the 851 participants primarily assigned to the intervention, control, or active groups renewed their consent in 2019, granting access to the data for an additional 5-year period. ^3^PA assessment may have been corrupted for the following reasons: medical conditions impairing normal physical behaviors during the assessment period (pregnancy, injury, etc.), acceleration signal summary not meeting inclusion criteria (4 days with a minimum of 10 h/d wearing time) despite several measurement attempts, etc. ^4^The number of analyzed data varies for each variable (see N values within brackets for each group). Reasons for missing data include: baseline assessment of physical activity not meeting quality standards, non-eligibility to physical fitness tests (e.g., participant becoming over 65 during the course of the study), impossible extraction of some specific parameters (e.g., vascular echo parameters due to the quality of images), participant not willing/able to perform one specific test on the date of the visit, among others

Participants engaged in the NEXIS participated in annual experimental visits at baseline and at +1- and +2-year (thereafter denoted, “+1-year follow-up” and “+2-year follow-up,” respectively). These visits included anthropometric measurements, physical fitness tests, blood examinations, vascular function tests, and dietary assessments. Prior to each visit, a 1-month objective assessment of PA was performed. The dietary assessment was performed using the brief self-administrative dietary habit questionnaire (BDHQ) [18]. These experimental visits occurred without discontinuation until March 2018. The results of all tests were sent to all the participants approximately 1 month after each visit. They included a description of the objective measurement of PA, as well as automatically generated dietary recommendations based on the BDHQ test outcomes. In this study, nine participants who presented corrupted data for the baseline PA were excluded from the analysis. The sample size of each group varied each year due to normal sample attrition over the study. Similarly, the size of analyzed samples varied for each variable for the following reasons: participant not able to perform some assessments due to a transient medical condition, participant not willing to perform some specific tests, and impossible extraction of some specific parameters, because the collected data did not meet the necessary quality standard. A CONSORT flow chart of the study is shown in Fig. 1. In the present analysis, MVPA was considered the primary outcome for assessing the effectiveness of the intervention.

### Objective evaluation of physical activity parameters

Physical activity was evaluated at baseline and +1 and +2-year follow-up using a waist-worn three-axis accelerometer monitor (Actimarker EW4800, Panasonic Electric Works, Osaka, Japan; dimensions, 74 × 33 × 13 mm; weight, 24 g). For 1 month, all participants were asked to wear the device continuously during the waking hours and remove it when going to bed and having a shower/bath and during water activities. During the evaluation periods, the screen of the monitor was always blinded, except for the one month prior to the +1-year follow-up evaluation of participants in the intervention group. For inclusion in analysis, an evaluation was required to have spanned a minimum of 14 days. If the first evaluation did not meet this criterion, the assessment was repeated.

The Actimarker EW4800 system samples the accelerometer sensor output at 20 Hz, and the signal is cleaned up using a low-pass filter. Computations involving the calculation of the three-dimensional vector norm every minute allows the synthesis and storage of the acceleration information at an epoch length of 1 min. Further algorithmic details related to the signal processing and the computation of PA parameters (i.e., prediction equations) can be found elsewhere [19–21]. Moreover, the system includes a peak-detection-based step-count algorithm. The hardware and software characteristics of the Actimarker EW4800 allow monitoring and storing of acceleration data for a continuous period of ≥ 30 days, without changing the battery or reset of memory. The validity of the Actimarker EW4800 for the objective assessment of PA has been demonstrated elsewhere [21, 22].

The present study reports the following PA parameters: step-count (steps/d), MVPA (MET-h/d), sedentary time (< 1.5 MET), and daily time of engaging in light PA (LPA, 1.5– 2.9 MET), moderate PA (MPA, 3.0–5.9 MET), ≥ 3 MET PA, ≥ 4 MET PA, ≥ 5 MET PA, and ≥ 6 MET PA (i.e., vigorous PA).

### Arms and intervention

Baseline MVPA and step-count values were used to split the participants into two groups. Participants performing at least 3.3 MET-h/d of MVPA and 10,000 steps/day were assigned to the “active” group. Participants not reaching 3.3 MET-h/d of MVPA or 10,000 steps/day were considered not optimally active with regard to the marks suggested by the Japanese “Exercise and Physical Activity Reference for Health Promotion 2006” [23], and were randomly split into two groups: “intervention” and “control.” Baseline characteristics of the participants are presented in Table 2.

A dynamic randomization algorithm was used to minimize the imbalance between the intervention and control groups for several stratification factors, such as sex, age, body mass index (BMI), PA, and location. The generation of the allocation sequence and assignment of participants to the control or intervention groups were outsourced to a third-party company (Mebix Ltd., Japan). Researchers were not involved in the allocation process. In order to ensure concealment of the group information, the study coordinators referred inactive participants to the company after the baseline evaluation of PA was completed. They coordinated the management of participants throughout the protocol without communicating the participant information to research staff during the experimental visits.

The objective of the PA intervention was to increase MVPA. The intervention included four interventional components, which had previously been given consideration for supporting PA increase in RCT or observational studies [24, 25]:

- Self-monitoring of PA using a waist-worn accelerometer-based device
- Physical behavior counseling
- Goal-setting
- Keeping a PA diary

In addition, the PA intervention comprised various characteristics, which can be described as follows:

- *Light*: Over the 1-year intervention period, the participants were assigned to five counseling sessions at intervals of 3 months, representing a total of 100–200 min of counseling.
- The objective of the PA intervention was to increase MVPA. The intervention included four interventional components, which had previously been given consideration for supporting PA increase in RCT or observational studies [24, 25]: Self-monitoring of PA using a waist-worn accelerometer-based device
- Physical behavior counseling
- Goal-setting
- Keeping a PA diary
  In addition, the PA intervention comprised various characteristics, which can be described as follows: *Light*: Over the 1-year intervention period, the participants were assigned to five counseling sessions at intervals of 3 months, representing a total of 100–200 min of counseling.
- *Oriented toward low-dose PA messages*: The participants were recommended to change their physical behaviors by adding small bouts of PA or by increasing the intensity of some selected activity of their daily routine (Table 1).
- *Progressive*: During the first one-on-one session, the counselor and the participant agreed on a 3-month target, which was reconsidered at each meeting, depending on the participant’s progress.
- *Individualized*: The sessions took the form of one-on-one counseling meetings, during which one certified counselor made recommendations tailored to the participant’s lifestyle.

**Table 1:** Examples of low-dose PA recommendations made during the counseling sessions to progressively increase MVPA

During daily life	Increase the weekly frequency of grocery shopping by buying fresh products and small portions each time you go to the supermarket Explore the supermarket hallways before starting your shopping Schedule a whole house cleaning session every week Add 10-min of walking when picking-up your child or friends at the bus stop/train station Do some stretching exercise during the TV commercials Walk faster during your utilitarian travels
During leisure time	Go to the gym with your family when you have a day off Registered to sport events in your local community Buy a fashionable pair of sneakers Play with your kids when accompanying them at the park
During working time	Take the stairs instead of the elevator when moving within your workplace Use the toilets upstairs Go out for lunch when possible
During commuting time	One time a week or more, stop one train station prior to the usual one and walk to your home Use the stairs instead of the elevator in the train station Board the train in the car located farthest to the station entrance/exit Walk faster during your commuting route Make a 10-min walk before using your car back home

Hereafter, the NEXIS intervention arm may be referred to by the expression “1-year PA intervention composed of brief counseling sessions and low-dose PA recommendations.” This expression is used for the sake of readability, and one should not omit all the above-mentioned characteristics of the intervention.

**Fig. 2:**
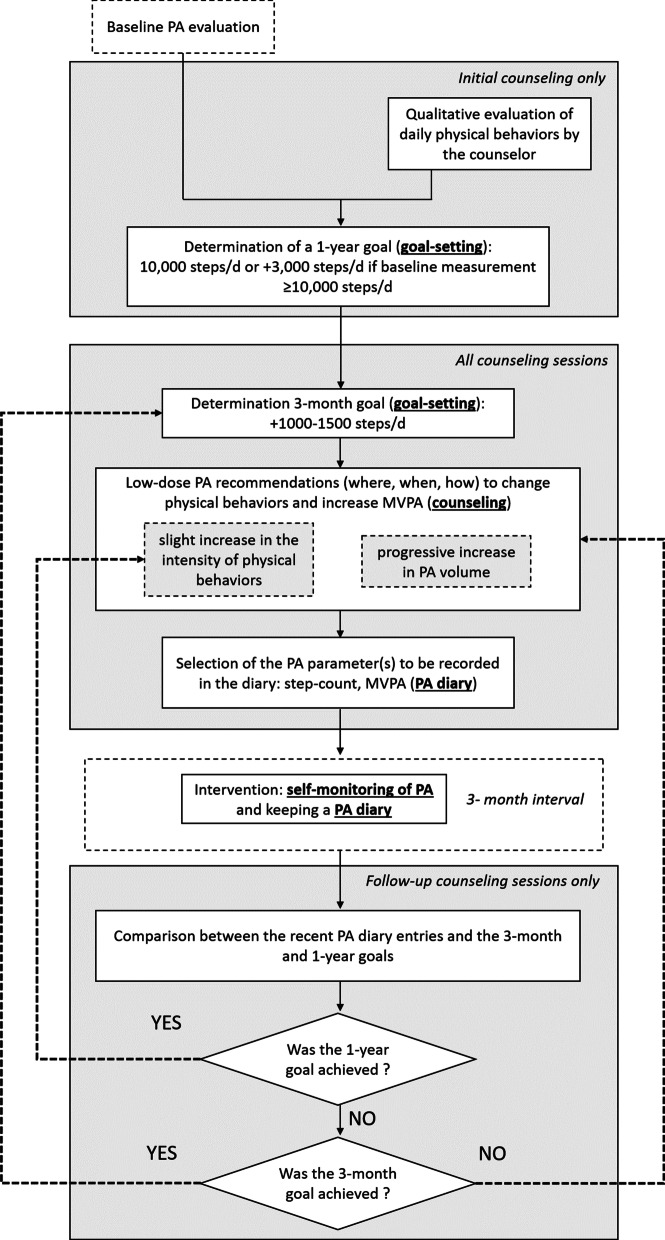
Structure of the PA intervention and flow of the information between the counseling sessions. The four components of the intervention are boldfaced and underlined

During the first counseling session, the counselor questioned the participant to obtain an accurate picture of her/his daily routines during weekdays and weekends to complement the outcome of the baseline objective PA assessment. The counselor and the participant agreed on a 1-year target. Participants presenting a baseline step-count < 10,000 steps per day were suggested to increase their PA until they could surpass this mark. Participants presenting a baseline step-count ≥ 10,000 steps per day were suggested to increase their daily PA by 3000 steps. In addition, a 3-month target corresponding to an increase of 1000–1500 steps/d was proposed for the participant; to reach this target, the participant was given individualized recommendations to change physical behaviors and progressively adopt a more active lifestyle. Places, moments of the day, and activities that could be used as platforms to increase the daily amount of MVPA were identified. As shown in Table 1, the recommendations made by the counselor mainly include low-dose PA messages. Despite the original targets being oriented toward an increase in the duration or frequency of active behaviors (1-year, 10,000 or +3000 steps/d; 3-month, +1000–1500 steps/d), the recommendations were tailored relative to the participant’s willingness and motivation, and could, therefore, include intensity-related content compatible with low-dose PA. For example, participants with limited amount of time to invest in PA could be advised to walk faster during their leisure or utilitarian travel to increase their MVPA levels without extending the time dedicated to PA. At the end of this first counseling session, the participant was given an Actimarker EW4800 monitor to wear every day during the intervention period. The participant was asked to record their daily step-count in a diary. Furthermore, the counseling session included exercise demonstrations when necessary. The four subsequent sessions had a similar structure. The 3-month target and recommendations on physical behavior change were adapted depending on the data recorded on the diary and the feedback provided by the patients. Participants who reached their 1-year target (10,000 or +3000 steps/d) before the end of the intervention period were suggested to increase MVPA through an increase in intensity rather than an increase in frequency or duration of activity bouts. Finally, some participants may have engaged in more vigorous forms of PA during the 1-year intervention. While the content of the counseling sessions aimed at supporting a progressive increase in MVPA by the means of low-dose PA messages, no upper limit was given to these participants. The overall structure of these sessions is presented in Fig. 2.

Participants in the intervention group wore the monitor every day for the entire 1-year period to complete their PA diary. However, data for the analyses were extracted from one month prior to the +1 year and +2 year follow-up visits, similar to the data extraction for the active and control groups. Participants assigned to the active and control groups were not given any direction with regard to PA and did not wear a monitor during the observation period except for one month prior to the visits (blind assessment). Moreover, no specific meeting or activity was carried out for these two groups during the course of the 2-year observation period.

### Nutrition, fitness, and health parameters

In addition to PA measurements, the NEXIS protocol evaluated dietary, health, and fitness parameters, most of which were considered as PA covariates. In the present analysis, the following parameters were selected in each category to be treated as dependent variables and investigate the potential impact of the PA intervention:

- *Body composition*: Weight, waist circumference.
- *Nutrition*: Total energy expenditure, protein intake, carbohydrate intake, and fat intake were evaluated using the BDHQ, which has been specifically developed for assessing dietary behaviors among the Japanese population [26].
- *Physical fitness*: Trunk flexibility, vertical jump, and peak oxygen consumption (VO_2_PEAK). Trunk flexibility was measured using a sit-and-reach digital instrument (T.K.K.5112; Takei Scientific Instruments Co., Ltd, Japan). The vertical jump test used a vertical jump measuring digital instrument (T.K.K.5406; Takei Scientific Instruments Co., Ltd, Japan). Measurement protocols were conducted in accordance with the manufacturer’s instructions. The VO_2_PEAK test was performed using an incremental cycle ergometer test and gas exchange analysis methods. The Douglas bag method (ARCO-1000; ARCO System, Chiba, Japan and DC-5; Shinagawa Corporation, Tokyo, Japan) was used for participants referred to the National Institutes of Biomedical Innovation, Health, and Nutrition in Tokyo, while breath-by-breath measurements were performed for participants referred to the Okayama Health Foundation, Okayama Prefecture (Oxycon Alpha, Jaeger, Würzburg, Germany). The cycle ergometer tests followed the same protocol in Tokyo and Okayama (start: 30 W, increment = 15 W/min).
- *Cardiovascular health*: Resting heart rate (HR), resting blood pressure, and resting brachial-ankle pulse wave velocity (baPWV) were evaluated using a plethysmography oscillometer system (Form PWV/ABI device, Omron Colin, Kyoto, Japan) according to the recommended method described elsewhere [27]. The blood pressure corresponds to the average of both the left and right arms mean arterial pressure. The baPWV was calculated by averaging the two brachial-ankle pulse wave velocities measured on the left and right sides of the body, respectively.
- *Blood markers*: Fasting glucose, glycosylated hemoglobin (Hb1Ac), triglycerides (TG), and total cholesterol were measured from blood samples using standard laboratory tests.

### Statistical analysis

First, differences between the intervention and active groups and those between the control and active groups were investigated at baseline for all selected dependent variables using a Welch analysis of variance (ANOVA) (Table 2).

**Table 2:** Participants’ characteristics and baseline values for physical activity covariates

	Active (N = 230)	Control (N = 161)	PA intervention (N = 183)
*Participant characteristics*			
Women/men	156/74	114/47	128/55
Age (year)	50 ± 9	48 ± 10	48 ± 9
BMI (kg/m^2^)	22.2 ± 2.6	22.2 ± 2.6	22.6 ± 3.2
Do/do not achieve 3.3 MET-h/d	230/0	39/122	45/138
Do/do not achieve 10,000 steps/d	230/0	21/140	23/160
*Nutrition parameters*			
Total energy intakes (kcal)	1854 ± 511	1787 ± 488	1775 ± 449
Protein intakes (g/1000 kcal)	38 ± 7	37 ± 6	37 ± 6
Carbohydrate intakes (g/1000 kcal)	133 ± 17	138 ± 17	136 ± 19
Fat intakes (g/1000 kcal)	28 ± 5	28 ± 6	29 ± 6
*Body composition*			
Weight (kg)	58 ± 10	58 ± 9	59 ± 10
Waist circumference (cm)	79 ± 9	80 ± 8	82 ± 9^†^
*Physical fitness parameters*			
Trunk flexibility (cm)	41 ± 8 (N = 229)	40 ± 11	40 ± 10 (N = 182)
Vertical jump (cm)	35 ± 10 (N = 224)	35 ± 10 (N = 159)	34 ± 10 (N = 177)
Peak oxygen consumption (mL/kg/min)	33 ± 7 (N = 222)*	30 ± 6 (N = 155)	30 ± 6 (N = 177)
*Cardiovascular parameters*			
Heart-rate (bpm)	61 ± 11*	65 ± 10	65 ± 13
Mean arterial pressure (mmHg)	90 ± 12	88 ± 11^†^	89 ± 12
baPWV (cm/s)	1228 ± 144	1229 ± 157	1236 ± 185
*Blood markers*			
Glucose (mg/dL)	90 ± 8	88 ± 9	90 ± 8
HbA1c (%)	5.3 ± 0.3	5.3 ± 0.3	5.3 ± 0.3
TG (mg/dL)	84 ± 45	90 ± 60	90 ± 49
Total cholesterol (mg/dL)	211 ± 33	207 ± 36	208 ± 33

Mean ± standard deviation. N = 230, 161, and 183 for the active, control, and low-dose PA intervention groups, respectively, except when otherwise noted

baPWV, brachial-ankle pulse wave velocity; BMI, body mass index; HbA1c, glycosylated hemoglobin; LPA, light physical activity; MPA, moderate physical activity; MVPA, moderate-to-vigorous physical activity; PA, physical activity; TG, triglyceride

^*^Different from the two other groups

^†^Different from the “active” group

**Fig. 3:**
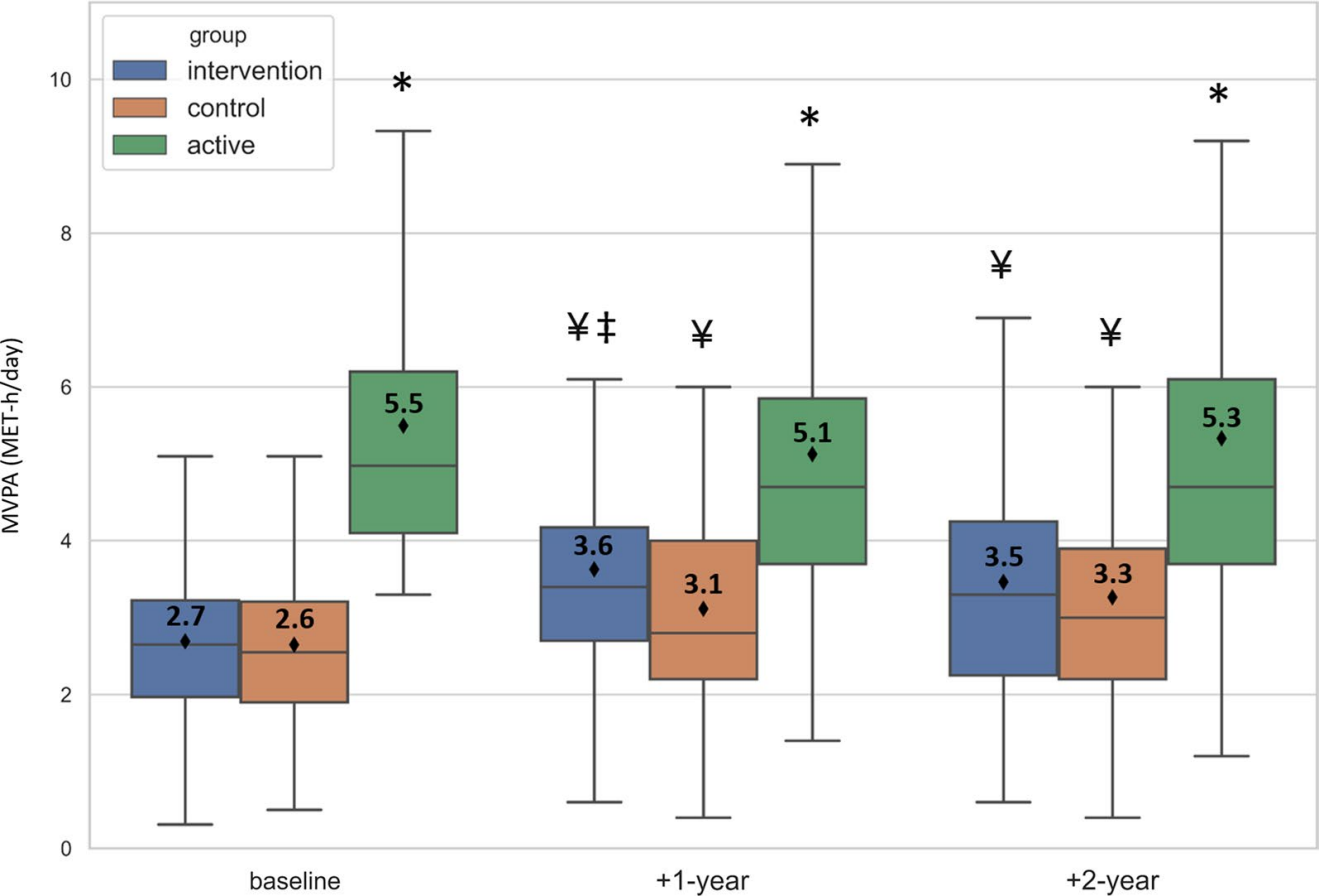
Moderate-to-vigorous physical activity in the active, control, and intervention groups over 3 years. The average value is indicated with a diamond mark (+ text) in each box. MVPA, moderate-to-vigorous physical activity. *Different from the two other groups. ^‡^Different from the control group. ^¥^Different from the baseline

Second, a mixed ANOVA test was used to investigate the changes in PA parameters over time and the differences between the intervention and control groups. Both per-protocol (PP) and intent-to-treat (ITT) analyses were conducted. The ITT treatment consisted of replacing the missing data with the baseline data of the same participant (Fig. 3, Table 3, and Additional file 1: Table S1). Post-hoc pairwise comparisons were conducted when an interaction effect between group and time factors was observed.

**Table 3:** Physical activity parameters in the active, control, and intervention groups over 3 years

	Group	Baseline	+1-year	+2-year	Interaction effect^1^		Baseline +1-year		Baseline +2-year
Step-count (steps/d)	Active	13,447 ± 3180 (N = 230)*	12,199 ± 3446 (N = 211)^¥^* [12265 ± 3379]	11,856 ± 3941 (N = 200)^¥^* [12138 ± 3981]	PP: *p* < 0.001 ITT: *p* < 0.001		PP: *p* = 0.001 ITT: *p* = 0.001		PP: *p* < 0.001 ITT: *p* < 0.001
	Control	8049 ± 2090 (N = 161)	8744 ± 2472 (N = 132) [8567 ± 2494]	8472 ± 3035 (N = 120) [8313 ± 2869]			PP: ns^3^ ITT: ns^3^		PP: ns ITT: ns
	PA intervention	8415 ± 1924 (N = 183)	9493 ± 2575 (N = 166) ^¥‡^ [9247 ± 2633]	8779 ± 2743 (N = 143) [8579 ± 2579]			PP: *p* < 0.001 ITT: *p* < 0.001		PP: ns ITT: ns
	RCT gr. effect ^2^	PP: ns ITT: ns	PP: *p* = 0.035 ITT: ns ^3^	PP: ns ITT: ns					
MVPA (MET-h/d)	Active	5.5 ± 2.1 (N = 230)*	5.1 ± 2.6 (N = 211)* [5.1 ± 2.5]	5.3 ± 3.2 (N = 200)* [5.4 ± 3.1]	PP: *p* < 0.001 ITT: *p* < 0.001		PP: ns ITT: ns	PP: ns ITT: ns	
	Control	2.6 ± 1.0 (N = 161)	3.1 ± 1.4 (N = 132)^¥^ [3.0 ± 1.4]	3.3 ± 1.9 (N = 120)^¥^ [3.1 ± 1.8]			PP: *p* = 0.01 ITT: *p* = 0.013		PP: *p* = 0.01 ITT: *p* = 0.013
	PA intervention	2.7 ± 1.1 (N = 183)	3.6 ± 1.8 (N = 166)^¥‡^ [3.5 ± 1.8]	3.5 ± 1.8 (N = 143)^¥^ [3.3 ± 1.7]			PP: *p* < 0.001 ITT: *p* < 0.001		PP: *p* < 0.001 ITT: *p* < 0.001
	RCT gr. effect ^2^	PP: ns ITT: ns	PP: *p* = 0.017 ITT: *p* = 0.017	PP: ns ITT: ns					
≥ 3 MET activity (min/d)	Active	83 ± 24 (N = 227)*	78 ± 30 (N = 211)* [78 ± 29]	80 ± 33 (N = 200)* [80 ± 33]	PP: *p* < 0.001 ITT: *p* < 0.001		PP: ns ITT: ns		PP: ns ITT: ns
	Control	43 ± 15 (N = 158)	50 ± 20 (N = 132)^¥^ [49 ± 20]	53 ± 28 (N = 120)^¥^ [50 ± 27]			PP: *p* = 0.005 ITT: *p* = 0.007		PP: *p* = 0.005 ITT: *p* = 0.006
	PA intervention	45 ± 16 (N = 181)	56 ± 22 (N = 166)^¥‡^ [55 ± 22]	55 ± 23 (N = 143)^¥^ [52 ± 23]			PP: *p* < 0.001 ITT: *p* < 0.001		PP: *p* < 0.001 ITT: *p* < 0.001
	RCT gr. effect ^2^	PP: ns ITT: ns	PP: *p* = 0.037 ITT: *p* = 0.035	PP: ns ITT: ns					
≥ 4 MET activity (min/d)	Active	27 ± 18 (N = 227)*	24 ± 20 (N = 211)* [24 ± 19]	26 ± 26 (N = 200)* [26 ± 25]	PP: *p* < 0.001 ITT: *p* < 0.001		PP: ns ITT: ns		PP: ns ITT: ns
	Control	9 ± 8 (N = 158)	11 ± 10 (N = 132) [11 ± 10]	12 ± 14 (N = 120) [12 ± 13]			PP: ns ITT: ns		PP: ns ITT: ns
	PA intervention	9 ± 7 (N = 181)	15 ± 14 (N = 166)^¥‡^ [15 ± 13]	14 ± 13 (N = 143)^¥^ [13 ± 12]			PP: *p* < 0.001 ITT: *p* < 0.001		PP: *p* < 0.001 ITT: *p* < 0.001
	RCT gr. effect ^2^	PP: ns ITT: ns	PP: *p* = 0.013 ITT: *p* = 0.012	PP: ns ITT: ns					
≥ 5 MET activity (min/d)	Active	9 ± 13 (N = 227)*	8 ± 14 (N = 211)* [8 ± 14]	10 ± 19 (N = 200)* [10 ± 19]	PP: *p* < 0.001 ITT: *p* < 0.001	PP: ns ITT: ns		PP: ns ITT: ns	
	Control	2 ± 4 (N = 158)	3 ± 5 (N = 132) [3 ± 5]	3 ± 6 (N = 120) [3 ± 6]		PP: ns ITT: ns		PP: ns ITT: ns	
	PA intervention	2 ± 3 (N = 181)	5 ± 8 (N = 166)^¥‡^ [4 ± 8]	4 ± 8 (N = 143)^¥^ [4 ± 7]		PP: *p* < 0.001 ITT: *p* < 0.001		PP: *p* = 0.009 ITT: *p* = 0.009	
	RCT gr. effect ^2^	PP: ns ITT: ns	PP: *p* = 0.019 ITT: *p* = 0.019	PP: ns ITT: ns					
≥ 6 MET activity (min/d)	Active	5 ± 10 (N = 227)*	5 ± 12 (N = 211) [5 ± 12]	6 ± 16 (N = 200)* [6 ± 16]	PP: *p* = 0.022 ITT: 0.017	PP: ns ITT: ns		PP: ns ITT: ns	
	Control	1 ± 2 (N = 158)	1 ± 4 (N = 132) [1 ± 4]	1 ± 4 (N = 120) [1 ± 4]		PP: ns ITT: ns		PP: ns ITT: ns	
	PA intervention	1 ± 1 (N = 181)	2 ± 7 (N = 166)^¥^ [2 ± 6]	2 ± 6 (N = 143) [2 ± 5]		PP: *p* = 0.008 ITT: *p* = 0.009		PP: ns^3^ ITT: ns^3^	
	RCT gr. effect ^2^	PP: ns ITT: ns	PP: ns ITT: ns	PP: ns ITT: ns					
Sedentary time (min/d)	Active	212 ± 48 (N = 227)^●^	215 ± 51 (N = 211) [214 ± 50]	218 ± 51 (N = 200) [217 ± 52]	PP: *p* < 0.001 ITT: *p* < 0.001	PP: ns ITT: ns		PP: ns ITT: ns	
	Control	224 ± 49 (N = 158)	222 ± 46 (N = 132) [220 ± 45]	222 ± 48 (N = 120) [221 ± 47]		PP: ns ITT: ns		PP: ns ITT: ns	
	PA intervention	227 ± 47 (N = 181)	218 ± 52 (N = 166) [220 ± 52]	224 ± 49 (N = 143) [223 ± 49]		PP: ns ITT: ns		PP: ns ITT: ns	
	RCT gr. effect ^2^	PP: ns ITT: ns	PP: ns ITT: ns	PP: ns ITT: ns					
LPA (min/d)	Active	365 ± 95 (N = 227)	364 ± 95 (N = 211) [362 ± 94]	369 ± 98 (N = 200) [369 ± 99]	PP: ns ITT: ns		PP: N/A ITT: N/A		PP: N/A ITT: N/A
	Control	341 ± 97 (N = 158)	361 ± 98 (N = 132) [352 ± 101]	353 ± 91 (N = 120) [347 ± 94]			PP: N/A ITT: N/A		PP: N/A ITT: N/A
	PA intervention	353 ± 96 (N = 181)	344 ± 101 (N = 166) [346 ± 98]	354 ± 96 (N = 143) [349 ± 96]			PP: N/A ITT: N/A		PP: N/A ITT: N/A
	RCT gr. effect ^2^	PP: N/A ITT: N/A	PP: N/A ITT: N/A	PP: N/A ITT: N/A					
MPA (min/d)	Active	78 ± 21 (N = 227)*	73 ± 25 (N = 211)* [73 ± 25]	74 ± 25 (N = 200)* [75 ± 25]	PP: *p* < 0.001 ITT: *p* < 0.001		PP: ns ITT: ns		PP: ns ITT: ns
	Control	42 ± 14 (N = 158)	49 ± 20 (N = 132)^¥^ [47 ± 19]	51 ± 26 (N = 120)^¥^ [49 ± 24]			PP: *p* = 0.005 ITT: *p* = 0.008		PP: *p* = 0.005 ITT: *p* = 0.006
	PA intervention	44 ± 16 (N = 181)	54 ± 19 (N = 166)^¥^ [52 ± 19]	53 ± 21 (N = 143)^¥^ [50 ± 21]			PP: *p* < 0.001 ITT: *p* < 0.001		PP: *p* = 0.001 ITT: *p* = 0.001
	RCT gr. effect ^2^	PP: ns ITT: ns	PP: ns^3^ ITT: ns^3^	PP: ns ITT: ns					

Mean ± SD ([] indicating the value for the intent-to-treat treatment)

gr., group; ITT, *p*-value for the intent-to-treat statistical treatment; LPA, light physical activity; MPA, moderate physical activity; MVPA, moderate-to-vigorous physical activity; N/A, non-applicable (for instance, when pairwise comparisons are not conducted due to the absence of interaction); Ns, non-significant; PA, physical activity; PP, *p*-value for the per-protocol statistical treatment; RCT, randomized controlled trial; “+1-year”: end of the intervention; “+2-year”: 1 year after the end of intervention

^1^Not reported in the table, a significant time effect has been observed for step-count, MVPA, ≥ 3 MET activity, ≥ 4 MET activity, ≥ 5 MET activity, ≥ 6 MET activity, MPA (increase) and sedentary time (decrease); in addition, a significant group effect was noted for step-count, MVPA, > 3 MET activity, ≥ 4 MET activity, ≥ 5 MET activity, ≥ 6 MET activity and MPA

^2^Refer to the difference between the PA intervention and the control group, i.e. not including the active group

^3^Trend (*p* < 0.01)

^*^Different from the two other groups

^●^Different from the intervention group

^‡^Different from the control group

^¥^Different from the baseline

Third, the same mixed ANOVA design was used to investigate the changes in dietary, health, and fitness parameters. Several significant interactions between the groups and the periods of measurement were revealed (Additional file 1: Table S1), and a Spearman correlation test was used to further investigate the relationship between the changes in these variables and the changes in MVPA, at +1-year and +2-year, in both the intervention and control groups (Table 4 and Additional file 2: Table S2, respectively). A Bonferroni correction was applied within each category of parameters: (1) nutrition, (2) body composition, (3) physical fitness, (4) cardiovascular, and (5) blood markers.

**Table 4:** Relationship between changes in PA and health parameters at 1- and 2-year (intervention group)

Change in MVPA vs.	Change in total energy intakes		Change in protein intakes		Change in carbohydrate intakes		Change in fat intakes	
Nutrition parameters	Baseline +1-year	Baseline +2-year	Baseline +1-year	Baseline +2-year	Baseline +1-year	Baseline +2-year	Baseline +1-year	Baseline +2-year
	R = 0.03 (ns)	R = 0.15 (ns)	R = 0.04 (ns)	R = − 0.02 (ns)	R = 0.07 (ns)	R = 0.05 (ns)	R = − 0.10 (ns)	R = − 0.08 (ns)

Change in MVPA vs.	Change in weight		Change in waist circumference	
Body composition parameters	Baseline +1-year	Baseline +2-year	Baseline +1-year	Baseline +2-year
	R = − 0.15 (ns)	R = − 0.05 (ns)	R = − 0.18 (ns)	R = − 0.08 (*p* = 0.036)

Change in MVPA vs.	Change in resting HR		Change in mean arterial pressure		Change in baPVW	
Vascular parameters	Baseline +1-year	Baseline +2-year	Baseline +1-year	Baseline +2-year	Baseline +1-year	Baseline +2-year
	R = − 0.22 (*p* = 0.015)	R = 0.00 (ns)	R = − 0.03 (ns)	R = 0.04 (ns)	R = − 0.07 (ns)	R = − 0.07 (ns)

Change in MVPA vs.	Change in blood glucose		Change in HbA1c		Change in TG		Change in total cholesterol	
Blood markers	Baseline +1-year	Baseline +2-year	Baseline +1-year	Baseline +2-year	Baseline +1-year	Baseline +2-year	Baseline +1-year	Baseline +2-year
	R = − 0.13 (ns)	R = − 0.18 (ns)	R = 0.02 (ns)	R = − 0.01 (ns)	R = 0.05 (ns)	R = − 0.01 (ns)	R = − 0.06 (ns)	R = 0.05 (ns)

Change in MVPA vs.	Change in trunk flexibility		Change in vertical jump height		Change in VO_2_PEAK	
Physical fitness parameters	Baseline +1-year	Baseline +2-year	Baseline +1-year	Baseline +2-year	Baseline +1-year	Baseline +2-year
	R = 0.03 (ns)	R = 0.04 (ns)	R = − 0.04 (ns)	R = − 0.03 (ns)	R = 0.15 (ns)	R = 0.23 (*p* = 0.030)

HbA1c, Glycosylated hemoglobin; HR, heart-rate; MVPA, moderate-to-vigorous physical activity; ns, non-significant; R, correlation coefficient; TG, Triglycerides; VO2PEAK, maximal oxygen consumption; “+1-year”: end of the intervention; “+2-year”: 1 year after the end of intervention. The results of the same analysis conducted in the control group are available as Additional file (Additional file 2: Table S2)

Finally, the participants in the intervention group were divided into subgroups relative to their sex, age group, and baseline level of MVPA, and mixed ANOVA tests were used to investigate the effect of participant characteristics on the intervention-induced change in PA. The same exploration was performed with the intervention group further split into the following tertiles: decrease or small increase in PA at the end of intervention versus moderate increase at the end of intervention versus large increase in PA at the end of intervention. Post hoc pairwise comparisons were conducted when an interaction effect between group and time factors was observed.

All analyses were performed using the open-source statistical package Pingouin v0.3.12 [28]. Results were expressed as the mean ± standard deviation. Values of the ITT treatment were indicated between square brackets, when necessary. *p* < 0.05 was considered significant.

## Results

### Physical activity parameters

As shown in Table 3, no difference was found between the intervention and control groups at baseline for any PA parameter, thus reflecting the validity of the randomization method for the group assignment process. The active group presented higher baseline values for step-count; MVPA; time spent in ≥ 3 MET, ≥ 4 MET, ≥ 5 MET, and ≥ 6 MET activities; and MPA (for both PP and ITT treatments, *p* < 0.001, except for ≥ 5 MET, *p* < 0.05 when compared with the intervention group).

A significant interaction between group and time factors was noted for step-count, MVPA, and for the time spent in ≥ 3 MET, ≥ 4 MET, ≥ 5 MET, and ≥ 6 MET and sedentary activities and MPA. Compared to baseline, a significant increase was noted at the end of the intervention (“1-year follow-up”) and 1 year after the end of the intervention (“2-year follow-up”) in the intervention group only, for the time spent in ≥ 4 MET and ≥ 5 MET activities, thus resulting in significantly higher values for the intervention group compared to the control group at +1-year. For example, a significant 6-min increase in the daily time spent in ≥ 4 MET activities was noted at +1-year in the intervention group. Similarly, the number of daily steps increased at +1-year in the intervention group only. However, the values returned to the baseline level at +2-year. Despite the interaction effect, the post hoc analysis did not allow locating any group or time difference for the “sedentary activity” parameter. In addition, a significant increase was found at +1-year and +2-year in both the intervention and control groups for MVPA and time spent in ≥ 3 MET activities. However, the effect was significantly more crucial in the intervention group, resulting in significantly higher values in the intervention group than in the control group at +1-year (p-values presented in Table 3). As shown in Fig. 3, the intervention group increased MVPA by 0.9 MET-h/d at +1-year compared to 0.5 MET-h/d for the control group. The active group presented higher values for step-count; MVPA; the time spent in ≥ 3 MET, ≥ 4 MET, ≥ 5 MET, and ≥ 6 MET; and MPA, consistently over 2 years of observation.

Among the 183 participants in the intervention group, the number of individuals reaching 3.3 MET-h/d of MVPA was altered as follows: 45, 95 (+ 111%), and 72 (+ 60%) at baseline, +1-year, and +2-year, respectively. For the 161 participants in the control group, the same variable evolved as follows: 39, 51 (+ 30%), and 48 (+ 23%), respectively. In addition, among the 183 participants in the intervention group, the number of individuals with over 10,000 steps/day was altered as follows: 23, 70 (+ 205%), and 42 (+ 82%) at baseline and +1- and +2-year follow-up, respectively. For the 161 participants in the control group, the same variable evolved as follows: 21, 46 (+ 119%), and 32 (+ 52%), respectively.

### Nutrition parameters

As shown in Table 2, no differences in daily total energy intake and adjusted protein, carbohydrate, and fat intake were found between the intervention and active groups, or between the control and active groups at baseline. A significant interaction between group and time factors was noted for the “total energy intakes” parameters (Additional file 1: Table S1). The intervention group presented significantly lower energy intakes than the control group at +2-year (1690 ± 430 vs. 1872 ± 565 kcal/d, *p* = 0.034 and 0.036, respectively, for the PP and ITT analyses). As shown in Table 4, no correlation was found between changes in MVPA and changes in nutrition parameters at +1-year and +2-year in the intervention group. Additional results related to the nutrition parameters are shown in Additional file 1: Table S1 and Additional file 2: Table S2.

### Health and fitness parameters

As shown in Table 2, the active group exhibited a lower resting HR and a higher VO_2_PEAK, whereas the intervention group presented a higher waist circumference compared to the active group. The control group had a significantly lower mean arterial pressure compared to that of the active group (90 ± 12 vs. 88 ± 11, *p* = 0.04). The active group presented a significantly lower resting HR compared to that of the control group at +1-year and +2-year (Additional file 1: Table S1). Fasting blood glucose concentrations decreased in both the active and intervention groups at +2-year.

In the intervention group, the changes in MVPA at +1-year were negatively correlated with the changes in resting HR (Table 4). In addition, the changes in MVPA at +2-year were negatively correlated with the changes in waist circumference and positively correlated with the change in VO_2_PEAK. In the control group, the changes in MVPA at +2-year tended to be correlated with the change in VO_2_PEAK (R = 0.22, *p* = 0.069). Additional results related to the health and fitness parameters are available in Tables S3 and S4.

### Factors influencing the intervention-induced changes in MVPA

Women assigned to the intervention group presented higher MVPA at baseline compared to their men counterparts. A time effect was noted in both groups at +1 and +2-year, thus eliminating the baseline sex difference. No age group effect was observed. A significant increase in MVPA was only noted in participants presenting a lower amount of MVPA at baseline (i.e., first and second tertiles in Table 5). However, the participants who were the most active at baseline (i.e., third tertile) still presented a statistically higher level of MVPA at +1- and +2-year.

**Table 5:** Intervention-induced changes in physical activity relative to selected participant characteristics

	Group	Baseline	+1-year	+2-year	Interaction effect ^1^	Baseline +1-year	Baseline +2-year
MVPA (MET-h/d) vs. sex	Women	2.9 ± 1 (N = 128)	3.6 ± 1.5 (N = 114)^¥^	3.4 ± 1.5 (N = 102)^¥^	*p* < 0.001	*p* = 0.001	*p* = 0.004
	Men	2.3 ± 1.1 (N = 55) ^Ø^(*p* = 0.005)	3.7 ± 2.4 (N = 52) ^¥^ ns	3.7 ± 1.5 (N = 41)^¥^ ns		*p* < 0.001	*p* = 0.010
MVPA (MET-h/d) vs. age group	30–39 year-old	2.6 ± 1.1 (N = 43)	3.3 ± 1.8 (N = 34)	3.1 ± 2.3 (N = 26)	ns	N/A	N/A
	40–49 year-old	2.6 ± 1.1 (N = 50)	4.0 ± 2.4 (N = 47)	3.8 ± 1.8 (N = 38)		N/A	N/A
	50–59 year-old	2.7 ± 1.0 (N = 67)	3.4 ± 1.3 (N = 63)	3.3 ± 1.5 (N = 58)		N/A	N/A
	60 year-old or more	3.2 ± 1.1 (N = 23)	4.1 ± 1.4 (N = 22)	3.7 ± 1.6 (N = 21)		N/A	N/A
MVPA (MET-h/d) vs. MVPA level at BL	Not active at BL (1st tertile)	1.6 ± 0.4 (N = 61) N/A	2.9 ± 2.3 (N = 52)^¥^	2.4 ± 1.7 (N = 44)^¥^	*p* = 0.003	*p* = 0.001	*p* = 0.011
	Moderately active at BL (2nd tertile)	2.6 ± 0.2 (N = 58) N/A	3.7 ± 1.4 (N = 54)^¥^	3.7 ± 1.8 (N = 47)^¥^ ^Ø^(vs. 1st tertile: *p* = 0.002)		*p* < 0.001	*p* = 0.001
	More active at BL (3rd tertile)	3.8 ± 0.7 (N = 64) N/A	4.2 ± 1.4 (N = 60) ^Ø^(vs. 1st tertile: *p* = 0.002)	4.1 ± 1.5 (N = 52) ^Ø^ (vs. 1st tertile: *p* < 0.001)		ns	ns

Mean ± SD. BL, baseline; N/A, non-applicable (for instance, when pairwise comparisons are not conducted due to the absence of interaction or when baseline MVPA values have been used to calculated tertiles); ns, non-significant; PA, physical activity; “+1-year”: end of the intervention; “+2-year”: 1 year after the end of intervention

^1^All the analyses output a significant time effect. A significant group effect was noted for the “vs. PA level at BL” analysis only

^¥^Different from the baseline

^Ø^A group effect (cf. details in the table)

As shown in Table 6, only participants with the highest increase in MVPA at the end of the intervention (third tertile) maintained a statistically higher level of MVPA compared to baseline values.

**Table 6:** Intervention-induced changes in physical activity relative to participant response at +1-year follow-up

	Group	Baseline	+1-year	+2-year	Interaction effect	Baseline +1-year	Baseline +2-year
MVPA (MET-h/d) vs. response to intervention	Decrease or small increase in PA at “+1-year”	3.0 ± 1.1 (N = 55) ns	2.5 ± 1.0 (N = 55)	2.8 ± 1.3 (N = 47)	*p* < 0.001	ns	ns
	Moderate increase in PA at “+1-year”	2.8 ± 1.0 (N = 54) ns	3.1 ± 1.1 (N = 54) ^Ø^(vs. the lowest tertile: *p* = 0.001)	3.3 ± 1.3 (N = 48)		ns	ns
	Large increase in PA at “+1-year”	2.5 ± 1.0 (N = 57) ns	5.0 ± 2.1 (N = 57)^¥^ ^Ø^(vs. the two other tertiles: *p* < 0.001)	4.5 ± 2.3 (N = 44)^¥^ ^Ø^(vs. the two other tertiles: *p* = 0.027 and *p* < 0.001, respectively)		*p* < 0.001	*p* < 0.001

Mean ± SD. ns, non-significant; PA, physical activity; “+1-year”: end of the intervention; “+2-year”: 1 year after the end of intervention. Significant group and time effects were noted

^¥^Different from baseline

^Ø^A group effect (cf. details in the table)

## Discussion

The most critical observation of this study was the significant increase in MVPA (0.9 MET-h/d) induced by the 1-year PA intervention, which was composed of five brief counseling sessions and oriented toward low-dose PA recommendations. The number of participants meeting the 3.3 MET-h standard increased by +111% in the intervention group versus 30% in the control group. Finally, active behaviors were retained by a significant number of the participants until 1 year after the intervention. The results of the study are discussed in a comprehensive manner in the following sections.

*Question #1*: Is a 1-year PA intervention composed of brief counseling sessions and low-dose PA recommendations effective in increasing the level of MVPA in a population of healthy Japanese adults?

Yes, to some extent.

The data analysis reports significant increases in a number of PA parameters in the PA intervention group. Interestingly, the time spent in ≥ 3 MET activities increased by 11 min on average, thus closely matching the volume increase that could be expected given the low-dose PA recommendations that were made by the counselors. Moreover, MVPA changed from a 2.7 ± 1.1 MET-h/d average at baseline to 3.6 ± 1.8 MET-h/d at +1-year, surpassing the 3.3 MET-h/d standard, which stands as the absolute target of the *Japanese official PA guidelines* [23, 29]. In addition, the increase in time spent in ≥ 4 MET and ≥ 5 MET activities was particularly tangible. This observation may reflect a change in walking habits, with participants in the intervention group actually adopting faster gait habits in response to one recurrent recommendation made during the counseling sessions: “please walk faster during your utilitarian travels/leisure activities” (Table 1). Therefore, recommending faster gait could be particularly relevant in the context of low-dose PA interventions to ensure that some adopted physical behaviors are “pushed” toward more intense activities.

The inclusion criteria used in the present study for the assignment of participants to the inactive pool were particularly restrictive (i.e., MVPA < 3.3 MET-h/d or step-count < 10,000 steps/d). Consequently, participants randomized to the intervention and control groups presented a relatively high level of activity (8244 ± 2002 steps/d vs. 6636 ± 4169 steps/d) on average in the Japanese population [30]. However, the PA intervention used in the present study showed promising outcomes, even in the least active participants. Fifty of the 138 participants who did not meet the 3.3 MET-h/d of MVPA at baseline did reach this standard at the end of the 1-year intervention period. In contrast, only 12 of the 122 control participants who did not reach the 3.3 MET-h/d of MVPA at baseline had reached this standard at +1-year.

Beyond these positive interpretations, one should not ignore that a significant part of participants did not respond to the intervention, or at least, did not overpass the 3.3 MET-h/d of MVPA. In addition, only 45% of the participants assigned to the PA intervention group (results not shown; ITT-treatment, 41%) presented a 10-min increase in ≥ 3 MET activities at the end of the 1-year intervention period. This outcome contrasts with that of the *National Health and Nutrition Survey of Japan* 2010, which indicated that 60% of Japanese adults were inclined to invest 10 min per day to increase their level of PA [31]. Therefore, the effectiveness of the intervention seems highly variable between individuals. More comprehensive research is needed to understand whether low-dose PA messages can remove psychological and physiological barriers to engage people, who were initially not inclined to adopt active behaviors or whether they act as a mere trigger for people, who would have responded to any type of intervention. Finally, the average daily step-count remained slightly under the 1-year target at the end of the intervention (9493 ± 2575 steps/d). According to feedback provided by the counselors, several participants chose to increase intensity when they had reached the 10,000 steps/day target before the end of the 1-year intervention (data not recorded). This might explain the more tangible increases observed for the time spent in ≥ 4 MET and ≥ 5 MET activities. This increase may have been achieved without a longer duration or higher frequency of activity bouts, thus limiting the increase in step-count.

*Question #2*: Are active behaviors possibly adopted during this type of PA intervention still observable 1 year after the end of the intervention?

Yes, at least for some participants.

One unique characteristic of the present study is the inclusion of objective PA assessments 1 year after the end of the intervention. From a public health perspective, long-term adherence to a PA program is a major determinant of its effectiveness. Indeed, it displays all subsequent physiological benefits [32, 33]. In the present analysis, the exploration of active behavior retention beyond the intervention period provides insight into the long-term adherence of the participants.

One year after the end of the intervention, the average MVPA remained significantly higher than the baseline level and higher than the 3.3 MET-h/d standard. The same observation was made for a number of other PA parameters including time spent in ≥ 3 MET (+ 10 min compared to baseline), ≥ 4 MET (+ 5 min), and ≥ 5 MET (+ 2 min) activities. In contrast with other PA studies, the physical behaviors of participants did not return to their baseline levels after the intervention [32]. The retention of active behaviors over such a long period may indicate good adherence of Japanese adults to a progressive program oriented toward low-dose PA messages. This result is particularly interesting given the “+10” top-line message of the current *Japanese official PA guidelines* [8].

However, further studies are needed to understand why some participants were not able to maintain MVPA over 3.3 MET-h/d for an additional year. One may hypothesize that some participants were simply not inclined to invest 10 min per day in PA, just like 40% of people who responded to the *National Health and Nutrition Survey of Japan* 2010 [31]. In addition, although not statistically significant, the time spent in sedentary and light PA tended to increase between +1- and +2-year, possibly indicating a return to less active behaviors for some participants. Overall, the parameters reflecting PA volume did not increase beyond a relatively low volume at +2-year, pointing to some possible limitations of low-dose PA recommendations, if the objective is to initiate a progressive increase toward more active behaviors in the long term.

*Question 3*: Is a 1-year PA intervention composed of brief counseling sessions and low-dose PA recommendations able to yield any significant physical fitness and health benefits?

No, or they were not observed over the course of the 2-year period of the study.

No statistical change was noted for any PA covariates at +1- and +2-year, except for blood glucose (Additional file 1: Table S1). However, this change occurred within the normal range and could not be considered of any physiological significance. Similarly, only a few significant correlations were found between the changes in MVPA and changes in health and fitness parameters in the intervention group (Table 4). Such observations were expected, since all health parameters were already in the normal range at the beginning of the study. Interestingly, significant correlations were noted for the change in MVPA on one side and the change in waist circumference, HR, and VO_2_PEAK on the other side. Related to the latter, a significant difference was observed between the active group and the intervention group for waist circumference and resting HR at baseline. The above observations suggest that a minimal increase in daily MVPA, i.e., that of +10-min or +1-MET-h, may still have the ability to improve parameters that tend to deviate from their optimal range in less-active people. However, these observations should be interpreted with caution given the small magnitude of the correlation coefficients or their proximity with the correlation coefficient observed in the control group for the same tests. Finally, it is unclear why the intervention group presented significantly lower energy intakes than the control group at +2-year (Additional file 1: Table S1). No time effect was noted in either the intervention or control group. One may suggest that participants allocated to the intervention group may have focused more on the various components of their lifestyle, including dietary intakes. However, the literature related to PA intervention, decrease in energy consumption, and appetite control is much contrasted, preventing any empirical interpretation [34]. Nevertheless, this result should be comprehended positively. Indeed, an increase in PA-related energy expenditures paired with either lower energy intakes or the preservation of pre-intervention energy intakes may produce protective effects relative to the risk of metabolic disorders.

Interventions using the low-dose PA concept have been used in pre-clinical populations; however, reports present contrasting results. In one study, a low-dose PA program has been ineffective in changing PA habits and reducing BMI in a population of obese adolescents [35]. In another study, a 1-year intervention resembling that of the NEXIS was associated with a > 4 kg decrease in a population of overweight Japanese adults [36]. The population of these two studies presented several differences with that of NEXIS; thus, one may still hypothesize that a long-term intervention could be a prerequisite to successful behavioral changes in the specific context of low-dose PA programs. In addition, modest improvements in biomarkers are usually noted in persons with cardiovascular risk factors or metabolic syndrome who participate in lifestyle counseling programs [37–39]. Regarding the specific case of healthy (yet inactive) participants, direct improvements of the health and fitness parameters may reasonably not be expected during a 2-year observation period. The primary objective of the NEXIS PA intervention arm was to increase MVPA in the most inactive people of the cohort. Positive effects on health parameters might therefore be observable in the long term, when the aging process exposes inactive people to the development of non-communicable diseases. One recent meta-analysis published by Miyachi et al. [15] and Ministry of Health, Labour, and Welfare [40] associated a 10-min increase in MVPA to a > 3% reduction in the relative risk for non-communicable diseases and mortality. The latter observation paired with the results of the present study emphasizes the relevance of including low-dose PA messages in public health policies aimed at promoting active behaviors in order to consolidate a minimal yet protective amount of PA within the healthy population, or at least a significant part of this population [9].

*Question #4*: Which factor influences the response to the PA intervention and the retention of the effect beyond the 1-year intervention period?

Answering this question would require further evidence.

In the present study, sex and age seem to impact neither the response to the intervention nor the retention of active behaviors at +2-year. No change in MVPA has been noted for the most-active participants, suggesting that an intervention composed of brief counseling sessions and low-dose PA recommendations would only be effective in the least-active people. In addition, only participants presenting the highest magnitude of MVPA change at +1-year were able to retain a higher amount of MVPA after 2 years, pointing toward the importance of intrinsic motivational factors for the adoption of active behaviors in the long term. However, the NEXIS protocol included only a few variables that could be used to explore factors influencing the success of the intervention. Therefore, the above observations should be challenged or confirmed in research protocols with a similar interventional setting; however, that includes the collection of variables able to detail the whole ecosystem of PA [41].

### Implementation of low-dose PA recommendations in real-world settings

Individual low-dose PA counselling approaches seem implementable in the current Japanese health care system. Individual lifestyle counselling sessions are already functional and covered by insurers for Japanese people aged 40–70 year who present metabolic syndrome risk factors. Each year, approximatively 1 million middle-aged Japanese people benefit from the Specific Health Guidance [39, 42]. While PA interventions (combined with diet recommendations or not) delivered through the Specific Health Guidance from 2008 to 2011 has been linked with significant but modest improvements in body compositions and cholesterol profiles; one may see the introduction of low-dose PA recommendations as an opportunity to yield better results through the engagement of most activity-resistant people. Further studies using the Specific Health Guidance platform and randomized controlled designs are, however, necessary to verify whether the results of the intervention presented in this manuscript can be observed in real-world settings for individual-level intervention.

At the collective level, the “Physical activity guidelines for the promotion of population health” released in 2013 has attempted to disseminate low-dose PA messages through the integration of the “+10” topline message [8, 10]. While objective measurements completed for National Health and Nutrition Survey of Japan before and after 2013 did not show any increase in the Japanese population daily PA [43], community wide interventions highlighting the “+10” message have been linked with a significant increase in self-reported PA in ≥ 65 year-old Japanese adults [44]. No significant change was noted in adults < 65 years old. One should note that the young and middle-aged adults enrolled in this study reported an average level of 85.3 min/day of PA at baseline. Drawing any conclusions on the effectiveness of low-dose PA message for increasing PA would therefore be premature without any study directed specifically to less active individuals. Further information on the penetration rate of the “+10” message among the Japanese population is also necessary to evaluate the impact of low-dose PA campaigns at the population level.

### Limitations and strengths

The present study had several limitations. First, significant increases in some PA parameters (MVPA and time spent in ≥ 3 MET activities) were found in the control group at +1- and +2-year. This could be explained by the recruitment method that may have attracted the attention of participants with intrinsic motivation for the adoption of a healthy lifestyle; the protocol design that provided annual feedback about the PA level to all participants engaged in the NEXIS; and a phenomenon of regression toward the mean observed in both the active and control groups. However, these potential confounding factors may not impact the analysis in a way that would invalidate the interpretation of the results. Indeed, the statistical analysis always revealed a larger size of the effect in the intervention group when a change in the control group was noted, and the change was only significant in the intervention group for step-count and the time spent in ≥ 4 MET and ≥ 5 MET activities. Moreover, the presence of these confounding factors may also allow optimistic interpretations. Despite the control group showing a significant increase in MVPA and time spent in ≥ 3 MET activities, the values for these two parameters were significantly higher in the intervention group at +1-year, suggesting the effectiveness of low-dose PA recommendations for PA in inactive people. Second, the NEXIS protocol included only one intervention arm, thus preventing any interpretation of the performance of a low-dose PA message compared to that of an intervention that would include challenging content, such as one of the WHO recommendations [7]. Finally, the participants of the study were recruited in two Japanese prefectures, and additional research is necessary before any generalization of the results to a non-Japanese population can be done.

In contrast, the present analysis had a few strengths. First, the NEXIS protocol was the first cohort study to include an intervention arm with healthy participants assigned to a prolonged and progressive PA intervention oriented toward low-dose PA recommendations. The “+10” message has now been disseminated to the Japanese population for up to 7 years, and the ActiveGuide document includes several guidance messages that are similar to the ones used by the NEXIS counselors [8]. These original results will be valuable for the evaluation of the current “Japanese official physical activity guidelines for the promotion of health,” especially since they will be revised in the upcoming years. Second, the protocol allowed for exploring the retention of behaviors 1 year beyond the strict period of intervention, a feature that is not frequently implemented in PA randomized control trials.

## Conclusions

In the present study, a 1-year exposure to low-dose PA recommendations aimed at progressively changing physical behaviors induced a significant increase in MVPA and helped raise PA toward the recommended standards in a population of healthy but not optimally active Japanese adults. Interestingly, a significant proportion of the participants retained active behaviors until 1 year after the intervention. The PA intervention did not impact the tested health parameters within the 2-year observation period of the study, and one may hope that the moderate change in MVPA would lower the risk of non-communicable diseases later in life, if it can be maintained for a longer period of time. Further studies are necessary to understand whether the inclusion of messages recommending a minimum amount of PA can be effective in initiating a progressive increase toward larger volumes of PA and to allow the best use of these messages in the context of individualized PA counseling programs as well as in broad public health campaigns.

## Supplementary Information

**Additional file 1: Table S1**. Nutrition, health, and fitness parameters in the active, control, and PA intervention groups over the 3 years of the study. baPWV, brachial-ankle pulse wave velocity; choles., cholesterol; circum., circumference; gr., group; ITT, *p*-value for the intent-to-treat statistical treatment; LPA, light physical activity; MPA, moderate physical activity; MVPA, moderate-to-vigorous physical activity; N/A, non-applicable (for instance, when pairwise comparisons are not conducted due to the absence of interaction); ns, non-significant; PA, physical activity; PP, *p*-value for the per-protocol statistical treatment; RCT, randomized controlled trial; TG, triglycerides; +1-year: end of the intervention; +2-year: 1 year after the end of intervention. ^1^Not reported in the table, a significant time effect has been observed for HR, blood glucose, TG (decrease), mean arterial pressure, baPWV, HbA1c, total cholesterol (increase). In addition, a significant group effect was noted for waist circumference, HR, VO2PEAK. ^2^Refer to the difference between the low-dose PA intervention and the control group, i.e. not including the active group. ^3^Trend (*p* < 0.1). ^4^Trend only for the ITT-treatment (*p* < 0.1). *Different from the two other groups (*p* < 0.05). ^#^Significantly different from the control group (*p* < 0.05).

**Additional file 2: Table S2**. [Table 4 counterpart for the control group] Relationship between the changes in physical activity and the change in health parameters at +1 and +2-year in the control group. HbA1c, Glycosylated hemoglobin; HR, heart-rate; MVPA, moderate-to-vigorous physical activity; ns: non-significant; R, correlation coefficient; TG, Triglycerides; VO2PEAK, maximal oxygen consumption; “+1-year”: end of the intervention; “+2-year”: 1 year after the end of intervention. ^1^Trend (*p* = 0.069).

**Additional file 3: Table S3**. Supporting data for “Materials and methods”, “Deviations from the registration”.

**Additional file 4: Table S4**. Incidence of lifestyle-related diseases (hypertension, dyslipidemia, diabetes) at baseline, +1-year follow-up and +2-year follow-up. Page 1: data for all participants. Page 2: data for the 453 participants who participated in the 3 visits.

**Additional file 5**. Raw data (baseline).

**Additional file 6**. Raw data (+1 year).

**Additional file 7**. Raw data (+2 years).

**Additional file 8**. Codebook for the categorical variables registered in the data set files.

## Data Availability

All data generated or analyzed during this study are included in the supplementary information files. Data are available in Additional files 5, 6, 7, 8.

## Abbreviations

ANOVA: Analysis of variance
baPWV: Brachial-ankle pulse wave velocity
BDHQ: Brief self-administrative dietary habit questionnaire
CONSORT: Consolidated standards of reporting trials
Hb1Ac: Glycosylated hemoglobin
HR: Heart rate
ITT: Intent-to-treat
LPA: Light physical activity
MET: Metabolic equivalent of task
MPA: Moderate physical activity
MVPA: Moderate-to-vigorous physical activity
NEXIS: Nutrition and exercise intervention study
PA: Physical activity
PP: Per-protocol
RCT: Randomized control trial
TG: Triglycerides
VO_2_PEAK: Peak oxygen consumption
WHO: World health organization
