# Supplementary material for: Effect of a 1-year intervention comprising brief counselling sessions and low-dose physical activity recommendations in Japanese adults, and retention of the effect at 2 years: a randomized trial
Source: BMC Sports Sci Med Rehabil. 2021 Oct 25;13:133. doi: 10.1186/s13102-021-00360-7 (PMC8543897; doi:10.1186/s13102-021-00360-7)
Supplement: Supplementary file 1 — Additional file 1: Table S1. Nutrition, health, and fitness parameters in the active, control, and PA intervention groups over the 3 years of the study. baPWV, brachial-ankle pulse wave velocity; choles., cholesterol; circum., circumference; gr., group; ITT, p-value for the intent-to-treat statistical treatment; LPA, light physical activity; MPA, moderate physical activity; MVPA, moderate-to-vigorous physical activity; N/A, non-applicable (for instance, when pairwise comparisons are not conducted due to the absence of interaction); ns, non-significant; PA, physical activity; PP, p-value for the per-protocol statistical treatment; RCT, randomized controlled trial; TG, triglycerides; +1-year: end of the intervention; +2-year: 1 year after the end of intervention. 1Not reported in the table, a significant time effect has been observed for HR, blood glucose, TG (decrease), mean arterial pressure, baPWV, HbA1c, total cholesterol (increase). In addition, a significant group effect was noted for waist circumference, HR, VO2PEAK. 2Refer to the difference between the low-dose PA intervention and the control group, i.e. not including the active group. 3Trend (p < 0.1). 4Trend only for the ITT-treatment (p < 0.1). *Different from the two other groups (p < 0.05). #Significantly different from the control group (p < 0.05). [file 13102_2021_360_MOESM1_ESM.docx]

**Additional file 1**

|  | Group | Baseline | +1-year follow-up | +2-year follow-up | Interaction effect ^1^ | Baseline + 1-year | | | Baseline +2-year |
| --- | --- | --- | --- | --- | --- | --- | --- | --- | --- |
| Energy intakes (kcal) | Active | 1854 ±511 (N=230) | 1824 ±468 (N=211)  [1843 ±503] | 1764 ±479 (N=202)  [1787 ±497] | PP: p<0.001  ITT: p<0.001 | PP: ns  ITT: ns | | | PP: ns  ITT: ns |
|  | Control | 1787 ±488 (N=161) | 1785 ±491 (N=133)  [1769 ±481] | 1872 ±565 (N=122)  [1819 ±549] |  | PP: ns  ITT: ns | | | PP: ns  ITT: ns |
|  | PA intervention | 1775 ± 449 (N=183) | 1726 ±433 (N=166)  [1729 ±435] | 1690 ±430 (N=144)  [1707 ±452] |  | PP: ns  ITT: ns | | | PP: ns  ITT: ns |
|  | RCT gr. effect ^2^ | PP: ns  ITT: ns | PP: ns  ITT: ns | PP: 0.036  ITT: 0.034 |  | | | | |
| Protein (/1000 kcal) | Active | 38 ±7 (N=230) | 39 ±6 (N=211)  [38 ±6] | 39 ±6 (N=202)  [39 ±6] | PP: ns ^3^  ITT: ns ^3^ | PP: N/A  ITT: N/A | | | PP: N/A  ITT: N/A |
|  | Control | 37 ±6 (N=161) | 38 ±6 (N=133)  [38 ±6] | 38 ±6 (N=122)  [38 ±6] |  | PP: N/A  ITT: N/A | | | PP: N/A  ITT: N/A |
|  | PA intervention | 37 ± 6 (N=183) | 38 ±6 (N=166)  [38 ±6] | 39 ±7 (N=144)  [39 ±7] |  | PP: N/A  ITT: N/A | | | PP: N/A  ITT: N/A |
|  | RCT gr. effect ^2^ | PP: N/A  ITT: N/A | PP: N/A  ITT: N/A | PP: N/A  ITT: N/A |  | | | | |
| Carbohydrates (/1000 kcal) | Active | 133 ±17 (N=230) | 134 ±17 (N=211)  [134 ±17] | 133±17 (N=202)  [133 ±17] | PP: ns ^3^  ITT: ns | PP: N/A  ITT: N/A | | | PP: N/A  ITT: N/A |
|  | Control | 138 ±17 (N=161) | 134 ±17 (N=133)  [135 ±17] | 136 ±17 (N=122)  [137 ±19] |  | PP: N/A  ITT: N/A | | | PP: N/A  ITT: N/A |
|  | PA intervention | 136 ± 19 (N=183) | 135 ±17 (N=166)  [135 ±17] | 134 ±19 (N=144)  [134 ±19] |  | PP: N/A  ITT: N/A | | | PP: N/A  ITT: N/A |
|  | RCT gr. effect ^2^ | PP: N/A  ITT: N/A | PP: N/A  ITT: N/A | PP: N/A  ITT: N/A |  | | | | |
| Fat (/1000 kcal) | Active | 28 ±5 (N=230) | 29 ±5 (N=211)  [29 ±5] | 29 ±5 (N=202)  [28 ±5] | PP: ns  ITT: ns | PP: N/A  ITT: N/A | PP: N/A  ITT: N/A | | |
|  | Control | 28 ±6 (N=161) | 29 ±5 (N=133)  [29 ±5] | 29 ±5 (N=122)  [29 ±6] |  | PP: N/A  ITT: N/A | | PP: N/A  ITT: N/A | |
|  | PA intervention | 29 ± 6 (N=183) | 29 ±6 (N=166)  [30 ±6] | 30 ±6 (N=144)  [30 ±6] |  | PP: N/A  ITT: N/A | | PP: N/A  ITT: N/A | |
|  | RCT gr. effect ^2^ | PP: N/A  ITT: N/A | PP: N/A  ITT: N/A | PP: N/A  ITT: N/A |  | | | | |
| Weight (kg) | Active | 58 ±10 (N=230) | 58 ±10 (N=212)  [58 ±10] | 57 ±10 (N=202)  [58 ±9] | PP: ns  ITT: ns | PP: N/A  ITT: N/A | | | PP: N/A  ITT: N/A |
|  | Control | 58 ±9 (N=161) | 59 ±9 (N=133)  [58 ±9] | 58 ±9 (N=122)  [58 ±9] |  | PP: N/A  ITT: N/A | | | PP: N/A  ITT: N/A |
|  | PA intervention | 59 ± 10 (N=183) | 59 ±10 (N=166)  [59 ±10] | 58 ±10 (N=144)  [59 ±10] |  | PP: N/A  ITT: N/A | | | PP: N/A  ITT: N/A |
|  | RCT gr. effect ^2^ | PP: N/A  ITT: N/A | PP: N/A  ITT: N/A | PP: N/A  ITT: N/A |  | | | | |
| Waist circum. (cm) | Active | 79 ±9 (N=230) | 79 ±8 (N=212)  [80 ±8] | 79 ±8 (N=202)  [80 ±8] | PP: ns  ITT: ns | PP: N/A  ITT: N/A | | | PP: N/A  ITT: N/A |
|  | Control | 80 ±8 (N=161) | 81 ±8 (N=133)  [81 ±8] | 81 ±8 (N=122)  [80 ±8] |  | PP: N/A  ITT: N/A | | | PP: N/A  ITT: N/A |
|  | PA intervention | 82 ± 9 (N=183) | 81 ±9 (N=166)  [81 ±9] | 79 ±8 (N=144)  [80 ±8] |  | PP: N/A  ITT: N/A | | | PP: N/A  ITT: N/A |
|  | RCT gr. effect ^2^ | PP: N/A  ITT: N/A | PP: N/A  ITT: N/A | PP: N/A  ITT: N/A |  | | | | |
| Heart rate (bpm) | Active | 61 ± 11 (N=230) * | 61 ± 10 (N=212) ^#^  [61 ±10] | 60 ± 11 (N=202) ^#4^  [61 ±11] | PP: p=0.007  ITT: p=0.010 | PP: ns  ITT: | | | PP: ns  ITT: |
|  | Control | 65 ± 10 (N=161) | 64 ± 9 (N=133)  [65 ±9] | 63 ± 9 (N=122)  [64 ±9] |  | PP: ns  ITT: ns | | | PP: ns  ITT: ns |
|  | PA intervention | 65 ± 13 (N=183) | 63 ± 11 (N=167)  [63 ±11] | 63 ± 12 (N=144)  [64 ±12] |  | PP: ns  ITT: ns | | | PP: ns  ITT: ns |
|  | RCT gr. effect ^2^ | PP: ns  ITT: ns | PP: ns  ITT: ns | PP: ns  ITT: ns |  | | | | |
| Mean arterial pressure (mmHg) | Active | 90 ± 12 (N=230) | 91 ± 13 (N=212)  [90 ±13] | 90 ± 12 (N=202)  [90 ±12] | PP: p<0.001  ITT: p<0.001 | PP: ns  ITT: ns | | | PP: ns  ITT: ns |
|  | Control | 88 ± 11 (N=161) | 89 ± 10 (N=133)  [89 ±11] | 89 ± 11 (N=122)  [88 ±12] |  | PP: ns  ITT: ns | | | PP: ns  ITT: ns |
|  | PA intervention | 89 ± 12 (N=183) | 89 ± 12 (N=167)  [89 ±12] | 90 ± 13 (N=144)  [90 ±12] |  | PP: ns  ITT: ns | | | PP: ns  ITT: ns |
|  | RCT gr. effect ^2^ | PP: ns  ITT: ns | PP: ns  ITT: ns | PP: ns  ITT: ns |  | | | | |
| baPWV (cm/s) | Active | 1228 ± 144 (N=230) | 1241 ± 163 (N=212)  [ 1236 ±160] | 1246 ± 157 (N=202)  [1242 ±157] | PP: p<0.001  ITT:  p<0.001 | PP: ns  ITT: ns | | | PP: ns  ITT: ns |
|  | Control | 1229 ± 157 (N=161) | 1227 ± 159 (N=133)  [1228 ±158] | 1249 ± 186 (N=122)  [1235 ±180] |  | PP: ns  ITT: ns | | | PP: ns  ITT: ns |
|  | PA intervention | 1236 ± 185 (N=183) | 1245 ± 165 (N=167)  [1238 ±170] | 1250 ± 178 (N=144)  [1240 ±177] |  | PP: ns  ITT: ns | | | PP: ns  ITT: ns |
|  | RCT gr. effect ^2^ | PP: ns  ITT: ns | PP: ns  ITT: ns | PP: ns  ITT: ns |  | | | | |
| Blood glucose  (mg/dL) | Active | 90 ± 8 (N=230) | 88 ± 9 (N=211)  [88 ±9] | 86 ± 9 (N=202)  [87 ±9] | PP: p=0.009  ITT: p=0.023 | PP: ns  ITT: ns | | | PP: p=0.003  ITT: p=0.003 |
|  | Control | 88 ± 10 (N=161) | 87 ± 10 (N=132)  [87 ±9] | 86 ± 9 (N=122)  [87 ±10] |  | PP: ns  ITT: ns | | | PP: ns  ITT: ns |
|  | PA intervention | 90 ± 8 (N=183) | 88 ± 9 (N=167)  [88 ±10] | 86 ± 8 (N=144)  [87 ±8] |  | PP: ns  ITT: ns | | | PP: p=0.002  ITT: p=0.004 |
|  | RCT gr. effect ^2^ | PP: ns  ITT: ns | PP: ns  ITT: ns | PP: ns  ITT: ns |  | | | | |
| HbA1c (%) | Active | 5.3 ± 0.3 (N=230) | 5.3 ± 0.4 (N=211)  [5.3 ±0.4] | 5.3 ± 0.3 (N=202)  [5.3 ±0.3] | PP: p<0.001  ITT: p<0.001 | PP: ns  ITT: ns | | | PP: ns  ITT: ns |
|  | Control | 5.3 ± 0.3 (N=161) | 5.3 ± 0.3 (N=132)  [5.3 ±0.3] | 5.4 ± 0.3 (N=122)  [5.4 ±0.3] |  | PP: ns  ITT: ns | | | PP: ns  ITT: ns |
|  | PA intervention | 5.3 ± 0.3 (N=183) | 5.3 ± 0.3 (N=167)  [5.3 ±0.3] | 5.4 ± 0.3 (N=144)  [5.4 ±0.3] |  | PP: ns  ITT: ns | | | PP: ns  ITT: ns |
|  | RCT gr. effect ^2^ | PP: ns  ITT: ns | PP: ns  ITT: ns | PP: ns  ITT: ns |  | | | | |
| TG (mg/dL) | Active | 84 ± 45 (N=230) | 87 ± 68 (N=212)  [86 ±66] | 82 ± 44 (N=202)  [83 ±44] | PP: p=0.020  ITT: p=0.043 | PP: ns  ITT: ns | | | PP: ns  ITT: ns |
|  | Control | 90 ± 60 (N=161) | 89 ± 57 (N=132)  [89 ±57] | 93 ± 70 (N=122)  [91 ±65] |  | PP: ns  ITT: ns | | | PP: ns  ITT: ns |
|  | PA intervention | 90 ± 49 (N=183) | 92 ± 52 (N=167)  [91 ±52] | 86 ± 50 (N=144)  [87 ±52] |  | PP: ns  ITT: ns | | | PP: ns  ITT: ns |
|  | RCT gr. effect ^2^ | PP: ns  ITT: ns | PP: ns  ITT: ns | PP: ns  ITT: ns |  | | | | |
| Total choles. (mg/dL) | Active | 211 ± 33 (N=230) | 215 ± 35 (N=212)  [213 ±34] | 213 ± 34 (N=202)  [214 ±34] | PP: p<0.001  ITT: p<0.001 | PP: ns  ITT: ns | | | PP: ns  ITT: ns |
|  | Control | 207 ± 36 (N=161) | 210 ± 35 (N=132)  [208 ±37] | 214 ± 38 (N=122)  [209 ±38] |  | PP: ns  ITT: ns | | | PP: ns  ITT: ns |
|  | PA intervention | 208 ± 33 (N=183) | 210 ± 32 (N=167)  [213 ±34] | 211 ± 32 (N=144)  [210 ±33] |  | PP: ns  ITT: ns | | | PP: ns  ITT: ns |
|  | RCT gr. effect ^2^ | PP: ns  ITT: ns | PP: ns  ITT: ns | PP: ns  ITT: ns |  | | | | |
| Trunk flexibility (cm) | Active | 41 ± 8 (N=229) | 41 ± 8 (N=211)  [41 ±8] | 41 ± 8 (N=201)  [41 ±8] | PP: ns  ITT: ns | PP: N/A  ITT: N/A | | | PP: N/A  ITT: N/A |
|  | Control | 40 ± 11 (N=161) | 40 ± 10 (N=133)  [40 ±11] | 39 ± 10 (N=122)  [40 ±11] |  | PP: N/A  ITT: N/A | | | PP: N/A  ITT: N/A |
|  | PA intervention | 40 ± 10 (N=182) | 41 ± 9 (N=165)  [41 ±9] | 40 ± 9 (N=142)  [40 ±9] |  | PP: N/A  ITT: N/A | | | PP: N/A  ITT: N/A |
|  | RCT gr. effect ^2^ | PP: N/A  ITT: N/A | PP: N/A  ITT: N/A | PP: N/A  ITT: N/A |  | | | | |
| Vertical jump (cm) | Active | 35 ± 10 (N=224) | 35 ± 10 (N=211)  [36 ±10] | 35 ± 10 (N=200)  [35 ±10] | PP: ns  ITT: ns | PP: N/A  ITT: N/A | | | PP: N/A  ITT: N/A |
|  | Control | 35 ± 10 (N=159) | 36 ± 9 (N=130)  [35 ±9] | 36 ± 9 (N=121)  [35 ±9] |  | PP: N/A  ITT: N/A | | | PP: N/A  ITT: N/A |
|  | PA intervention | 34 ± 10 (N=177) | 35 ± 10 (N=159)  [35 ±10] | 34 ± 10 (N=142)  [34 ±10] |  | PP: N/A  ITT: N/A | | | PP: N/A  ITT: N/A |
|  | RCT gr. effect ^2^ | PP: N/A  ITT: N/A | PP: N/A  ITT: N/A | PP: N/A  ITT: N/A |  | | | | |
| Peak Oxygen consumption (mL/kg /min) | Active | 33.3 ± 7.2 (N=222) | 33.0 ± 7.6 (N=210)  [33 ±7.5] | 33.0 ± 7.8 (N=193)  [33.2 ±7.7] | PP: ns  ITT: ns | PP: N/A  ITT: N/A | | | PP: N/A  ITT: N/A |
|  | Control | 30.0 ± 6.2 (N=155) | 29.7 ± 6.9 (N=129)  [29.3 ±6.7] | 30.6 ± 7.2 (N=112)  [29.9 ±6.8] |  | PP: N/A  ITT: N/A | | | PP: N/A  ITT: N/A |
|  | PA intervention | 29.8 ± 6.4 (N=177) | 30.8 ± 7.0 (N=160)  [30.4 ±6.9] | 30.7 ± 7.8 (N=132)  [30.2 ±6.8] |  | PP: N/A  ITT: N/A | | | PP: N/A  ITT: N/A |
|  | RCT gr. effect ^2^ | PP: N/A  ITT: N/A | PP: N/A  ITT: N/A | PP: N/A  ITT: N/A |  | | | | |
