# Supplementary material for: Effect of a 1-year intervention comprising brief counselling sessions and low-dose physical activity recommendations in Japanese adults, and retention of the effect at 2 years: a randomized trial
Source: BMC Sports Sci Med Rehabil. 2021 Oct 25;13:133. doi: 10.1186/s13102-021-00360-7 (PMC8543897; doi:10.1186/s13102-021-00360-7)
Supplement: Supplementary file 2 — Additional file 2: Table S2. [Table 4 counterpart for the control group] Relationship between the changes in physical activity and the change in health parameters at +1 and +2-year in the control group. HbA1c, Glycosylated hemoglobin; HR, heart-rate; MVPA, moderate-to-vigorous physical activity; ns: non-significant; R, correlation coefficient; TG, Triglycerides; VO2PEAK, maximal oxygen consumption; “+1-year”: end of the intervention; “+2-year”: 1 year after the end of intervention. 1Trend (p = 0.069). [file 13102_2021_360_MOESM2_ESM.docx]

**Additional file 2: Table S2. [Table 4 counterpart for the control group] Relationship between the changes in physical activity and the change in health parameters at + 1 and + 2-year in the control group**

| Change in MVPA vs. | Change in total energy intakes | | Change in protein intakes | | Change in carbohydrate intakes | | Change in fat intakes | |
| --- | --- | --- | --- | --- | --- | --- | --- | --- |
| Nutrition parameters | Baseline  + 1-year | Baseline  +2-year | Baseline  + 1-year | Baseline  +2-year | Baseline  + 1-year | Baseline  +2-year | Baseline  + 1-year | Baseline  +2-year |
|  | R = 0.05  (ns) | R = −0.041  (ns) | R = 0.029  (ns) | R = −0.109  (ns) | R = −0.04  (ns) | R = 0.23  (p=0.044) | R = −0.06  (ns) | R = −0.12  (ns) |
| Change in MVPA vs. | Change in weight | | Change in waist circumference | |  | |  | |
| Body composition parameters | Baseline  + 1-year | Baseline  +2-year | Baseline  + 1-year | Baseline  +2-year |  |  |  |  |
|  | R = −0.10  (ns) | R = −0.01  (ns) | R = 0.06  (ns) | R = −0.02  (ns) |  |  |  |  |
| Change in MVPA vs. | Change in resting HR | | Change in mean arterial pressure | | Change in baPVW | |  | |
| Vascular parameters | Baseline  + 1-year | Baseline  +2-year | Baseline  + 1-year | Baseline  +2-year | Baseline  + 1-year | Baseline  +2-year |  |  |
|  | R = −0.05  (ns) | R = −0.15  (ns) | R = −0.01  (ns) | R = 0.01  (ns) | R = −0.04  (ns) | R = −0.07  (ns) |  |  |
| Change in MVPA vs. | Change in blood glucose | | Change in HbA1c | | Change in TG | | Change in total cholesterol | |
| Blood markers | Baseline  + 1-year | Baseline  +2-year | Baseline  + 1-year | Baseline  +2-year | Baseline  + 1-year | Baseline  +2-year | Baseline  + 1-year | Baseline  +2-year |
|  | R = 0.06  (ns) | R = 0.11  (ns) | R = 0.03  (ns) | R = −0.04  (ns) | R = −0.03  (ns) | R = −0.08  (ns) | R = −0.08  (ns) | R = −0.01  (ns) |

| Change in MVPA vs. | Change in trunk flexibility | | Change in vertical jump height | | Change in VO_2_PEAK | |  |
| --- | --- | --- | --- | --- | --- | --- | --- |
| Physical fitness parameters | Baseline  + 1-year | Baseline  +2-year | Baseline  + 1-year | Baseline  +2-year | Baseline  + 1-year | Baseline  +2-year |  |
|  | R = −0.03  (ns) | R = 0.19  (ns) ^1^ | R = 0.09  (ns) | R = −0.04  (ns) | R = 0.07  (ns) | R = 0.22  (ns) ^1^ |  |

HbA1c: Glycosylated hemoglobin; HR: heart-rate; MVPA: moderate-to-vigorous physical activity; ns: non-significant; R: correlation coefficient; TG: Triglycerides; VO2PEAK: maximal oxygen consumption; “+ 1-year”: end of the intervention; “+ 2-year”: 1 year after the end of intervention. ^1^: trend (p = 0.069).
