# Supplementary material for: Effect of a 1-year intervention comprising brief counselling sessions and low-dose physical activity recommendations in Japanese adults, and retention of the effect at 2 years: a randomized trial
Source: BMC Sports Sci Med Rehabil. 2021 Oct 25;13:133. doi: 10.1186/s13102-021-00360-7 (PMC8543897; doi:10.1186/s13102-021-00360-7)
Supplement: Supplementary file 4 — Additional file 4: Table S4. Incidence of lifestyle-related diseases (hypertension, dyslipidemia, diabetes) at baseline, +1-year follow-up and +2-year follow-up. Page 1: data for all participants. Page 2: data for the 453 participants who participated in the 3 visits. [file 13102_2021_360_MOESM4_ESM.docx]

**Additional file 4**

| **Hypertension:** | | | |
| --- | --- | --- | --- |
| Group: | Baseline | +1-year | +2-year |
| Active | 8 / 230 | 19 / 212 | 15 / 202 |
| Control | 12 / 161 | 9 / 133 | 10 / 122 |
| Intervention | 13 / 183 | 16 / 167 | 17 / 145 |

| **Dyslipidemia:** | | | |
| --- | --- | --- | --- |
| Group: | Baseline | +1-year | +2-year |
| Active | 22 / 230 | 28 / 212 | 26 / 202 |
| Control | 14 / 161 | 16 / 133 | 14 / 122 |
| Intervention | 14 / 183 | 15 / 167 | 20 / 145 |

| **Diabetes:** | | | |
| --- | --- | --- | --- |
| Group: | Baseline | +1-year | +2-year |
| Active | 3 /230 | 4 / 212 | 2 / 202 |
| Control | 1 / 161 | 2 / 133 | 1 / 122 |
| Intervention | 1 / 183 | 2 / 167 | 0 / 145 |

| **Hypertension:** | | | |
| --- | --- | --- | --- |
| Group: | Baseline | +1-year | +2-year |
| Active | 8 / 194 | 18 / 194 | 15 / 194 |
| Control | 8 / 117 | 8 / 117 | 8 / 117 |
| Intervention | 11 / 142 | 14 / 142 | 16 / 142 |

| **Dyslipidemia:** | | | |
| --- | --- | --- | --- |
| Group: | Baseline | +1-year | +2-year |
| Active | 21 / 194 | 27 / 194 | 26 / 194 |
| Control | 13 / 117 | 16 / 117 | 14 / 117 |
| Intervention | 9 / 142 | 11 / 142 | 20 / 142 |

| **Diabetes:** | | | |
| --- | --- | --- | --- |
| Group: | Baseline | +1-year | +2-year |
| Active | 3 / 194 | 4 / 194 | 2 / 194 |
| Control | 1 / 117 | 1 / 117 | 1 / 117 |
| Intervention | 0 / 142 | 2 / 142 | 0 / 142 |
